# Quantitative PCR provides a simple and accessible method for quantitative microbiota profiling

**DOI:** 10.1371/journal.pone.0227285

**Published:** 2020-01-15

**Authors:** Ching Jian, Panu Luukkonen, Hannele Yki-Järvinen, Anne Salonen, Katri Korpela

**Affiliations:** 1 Human Microbiome Research Program, Faculty of Medicine, University of Helsinki, Helsinki, Finland; 2 Minerva Foundation Institute for Medical Research, Helsinki, Finland; 3 Department of Medicine, University of Helsinki and Helsinki University Central Hospital, Helsinki, Finland; Universidade Catolica Portuguesa, PORTUGAL

## Abstract

The use of relative abundance data from next generation sequencing (NGS) can lead to misinterpretations of microbial community structures, as the increase of one taxon leads to the concurrent decrease of the other(s) in compositional data. Although different DNA- and cell-based methods as well as statistical approaches have been developed to overcome the compositionality problem, and the biological relevance of absolute bacterial abundances has been demonstrated, the human microbiome research has not yet adopted these methods, likely due to feasibility issues. Here, we describe how quantitative PCR (qPCR) done in parallel to NGS library preparation provides an accurate estimation of absolute taxon abundances from NGS data and hence provides an attainable solution to compositionality in high-throughput microbiome analyses. The advantages and potential challenges of the method are also discussed.

## Introduction

**Fig 1:**
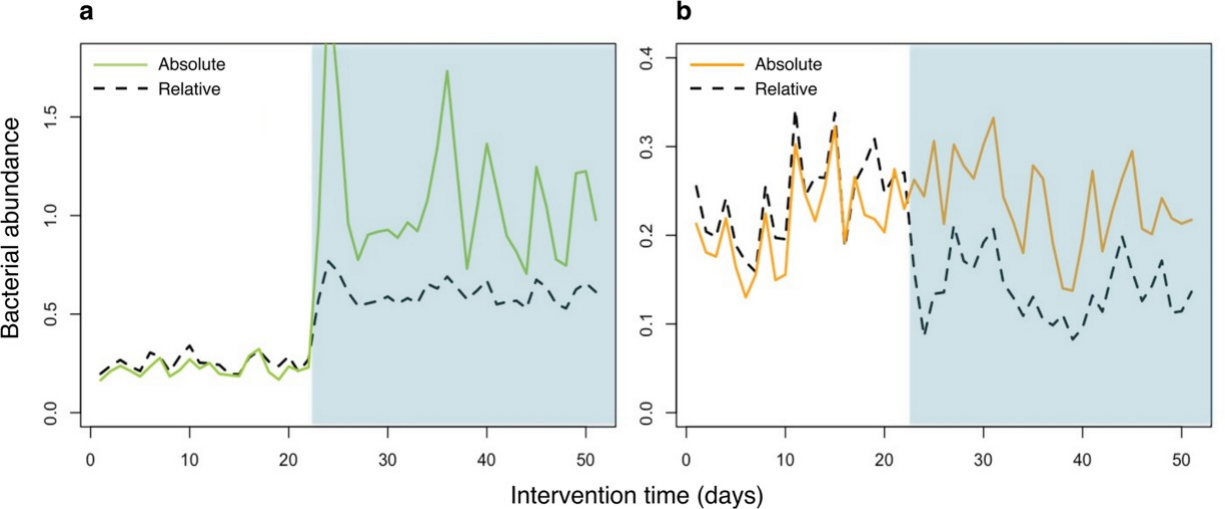
Compositionality leading to false positive discoveries. To demonstrate the effect of compositionality on interpretation of microbiome NGS data, an intervention was simulated where a single taxon increased in abundance. The simulation was conducted in absolute abundance, which was converted to relative abundance for data analysis. (a) The intervention (shaded area) increased a single taxon (green solid line), which remained true when converted to relative abundance (black dashed line). (b) Other taxa (a single taxon represented here by the orange solid line) were not affected by the intervention. However, the relative abundance (black dashed line) shows a negative impact of the intervention, due to the increase in relative abundance of the affected taxon in (a).

The use of relative abundance from next generation sequencing (NGS) data can lead to misinterpretations of microbial community structures as due to compositionality, the relative abundances of the taxa being mutually dependent. This means that an increase of one taxon inevitably leads to the concurrent decrease of the other(s). Since the changes of components are mutually dependent, high false discovery rates occur when compositional data are analyzed using traditional statistical methods [1]. Correlation analysis of relative abundance data is strongly subject to a negative correlation bias and spurious associations [2]. Meanwhile, compositionality particularly hampers the interpretation of microbial changes in longitudinal studies, such as interventions. Without NGS-independent experiments as validation, it is problematic to determine which taxon was truly affected by an intervention, i.e. to identify the actual target organism(s) for a specific treatment (Fig 1).

Contrary to the speculation that compositionality is dismissible in high complexity environments [3], our simulations revealed that the compositionality effects may lead to extensive false positive findings in both complex microbial communities (e.g., gut) as well as samples with low diversity (e.g., vaginal swab) (S1 and S2 Figs). Sophisticated statistical methods have been developed in an effort to mitigate the effect of mutual dependence of component changes in compositional sequencing data [1–4]. However, unknown absolute abundances cannot be deduced from compositional data using statistical methods, yet recent studies have shown that absolute abundances of bacteria are biologically meaningful [5–7]. Taken together, relying solely on relative abundance results in false findings (Fig 1) as well as the omission of important information on the interactions of different taxa with each other and the host [8].

Absolute quantification of microbial abundances from NGS datasets (i.e. quantitative microbiome profiling) can be achieved by integrating cell-based or DNA-based methods into standard NGS workflows. Flow cytometry has been applied to complement amplicon sequencing in an engineered freshwater ecosystem [9] and recently by Vandeputte et al. [6] for fecal samples. For DNA-based methods, spike-in bacteria [10], synthetic DNA [11] as well as quantitative PCR (qPCR) have been employed to estimate NGS-derived absolute abundances of penile microbiota [7] and environmental fungi [12]. Recently, DNA yield was used to document quantitative variations in the fecal microbiota of numerous mammalian species as well as in human patients after fecal microbiota transplant [5], and to investigate microbiota development in premature infants [13]. Notwithstanding the variety of methods that have been introduced to overcome artefacts related to data compositionality, they have not been adopted for the human microbiome research. Of note, none of the clinical trials published during the past year (except the ones specifically addressing the compositionality problem [5, 6]) utilized quantitative microbiome profiling. Here, we present how quantitative PCR (qPCR)-based bacterial enumeration can be integrated to NGS pipelines to provide a feasible approach to estimate absolute abundances from NGS data, and hence promote the use of quantitative microbiome profiling in the field of human microbiome.

## Materials and methods

### Study subjects and fecal sample collection

The study used the samples derived from an intervention registered at ClinicalTrials.gov as NCT02133144. The study protocol was approved by the Medical Ethical Committees of the Hospital District of Helsinki and Uusimaa and Helsinki University Central Hospital. All volunteers provided an informed, written consent. The study cohort consisting of 38 adult human subjects has been described previously [14]. The trial aimed to study the metabolic effects of hypercaloric diets enriched in different macronutrients. The study protocol was approved by the Medical Ethical Committees of the Hospital District of Helsinki and Uusimaa and HUCH. For the current study, we additionally included follow-up samples collected after the trial, amounting to a total of 114 samples. Fecal samples were self-collected and stored at -20°C, and then transferred to the long-term storage at -80°C within 1 day.

### Bacterial DNA extraction

Bacterial DNA was extracted from fecal samples using a modified version of repeated bead beating [15] that efficiently extracts bacterial DNA from both Gram-positive and -negative cocci [16]. Briefly, immediately after thawing, 0.125 g of feces were weighted and added into 2.0 ml screw-up tubes pre-filled with 0.25 g of 0.1 mm zirconia beads and 3 of 3 mm glass beads. Fecal samples were re-suspended to 0.5 ml of lysis buffer (500 mM NaCl, 50 mM Tris-HCL (pH 8), 50 mM EDTA, 4% SDS). Two successive rounds of 1-minute bead beating were done using a FastPrep^®^-24 instrument (MP Biomedicals, Santa Ana, CA, USA) at 5.5 m/s. The lysate fraction produced from the first round of bead beating was collected before the second round to minimize DNA shearing [15]. Each round of bead beating was followed by a 15-min incubation period at 95°C to further enhance the lysis. After precipitation of DNA, the DNA was further purified by using the QIAamp DNA Mini Kit columns (Qiagen, Hilden, Germany). The purified DNA was quantified for DNA concentration using a Qubit^®^ fluorometer (Invitrogen, CA, USA) before storing at -20° C until further use. All the fecal samples were processed within 10 days.

### 16S rRNA gene sequencing

Illumina MiSeq paired-end sequencing of the hypervariable V3-V4 regions of the 16S rRNA gene (primers 341F/785R) was performed according to the manual from Illumina with a slight modification where dual index TrusSeq-tailed 1-step amplification [17] was used for library preparation. The detailed protocol for library preparation has been described [14]. The pooled libraries were sequenced with an Illumina MiSeq instrument using paired end 2 × 300 bp reads and a MiSeq v3 reagent kit with 5% PhiX as spike-in. The sequencing was carried out at the sequencing unit of the Institute for Molecular Medicine Finland (FIMM), Helsinki, Finland.

### Sequencing data processing and analysis

The preprocessing was done in the R package *mare* [18], utilizing USERACH for quality filtering, chimera removal, and taxonomic annotation [19]. Only the high-quality forward reads were used, as we have previously shown that this approach provides the most accurate results [13]. The forward reads were truncated to length of 150 bases with *mare*’s “ProcessReads” command. We used default settings for minimum quality score (2) and maximum expected errors (1). Reads with prevalence below 0.01% were removed, as they are likely to contain errors. To avoid potential biases in taxonomic annotation caused by OTU clustering [20], truncated, filtered and dereplicated reads were directly annotated using the Silva 115 database [21], restricted to gut-associated taxa as done in our previous studies [13, 22].

### Quantitative PCR

Quantification of total bacteria, specific taxa and butyrate production capacity was carried out by qPCR using a BioRad iCycler iQ thermal cycler system (BioRad, Hercules, CA) with HOT FIREPol^®^ EvaGreen^®^ qPCR Mix Plus (Solis BioDyne, Tartu, Estonia). The 331F/797R primers were chosen for the quantification of total bacteria, as the primers target the V3-V4 hypervariable regions as in Illumina MiSeq. A list of primers and references used in the present study is summarized in S1 Table.

For bacterial enumeration, total bacteria, *Clostridium* cluster XIVa and Bacteroidetes were quantified using 0.5 ng of fecal DNA, for the less abundant *Bifidobacterium* and *E*. *coli* groups 25 ng DNA/reaction was used. Detailed information on the PCR conditions has been described previously [15, 23]. Briefly, the thermal cycling conditions started with a DNA-denaturation step at 95° C for 15 minutes, followed by 40 cycles of 1) denaturation at 95° C for 15 seconds, 2) annealing at a primer-specific temperature (Annealing (°C) in S1A Table) for 20 seconds, 3) extension at 72° C for 30 seconds and 4) an incubation step at a primer-specific temperature to detect the fluorescent data (Detection (°C) in S1A Table). A melting curve analysis was carried out to ensure the specificity of the amplification products. The 10-log-fold standard curves ranging from 10^2^ to 10^7^ copies were produced using the full-length amplicons of 16S rRNA gene of appropriate reference organisms [23] (*Ruminococcus productus* for *Clostridium* cluster XIVa, *Bacteroides fragilis* for Bacteroidetes, *Bifidobacterium longum* for *Bifidobacterium*/total bacteria, and *Escherichia coli DSM 6897* for *E*. *coli*) to convert the threshold cycle (Ct) values into the average estimates of target bacterial genomes present in 1 g of feces (copy numbers/g of wet feces) in each assay.

For quantification of butyrate production capacity of the microbiota, the butyryl-CoA:acetate CoA-transferase gene was quantified by qPCR as described [24], and the output values were converted based on comparative Ct method [25]. The results were correlated to the NGS-based abundance of the dominant butyrate-producing genera *Subdoligranulum*, *Faecalibacterium*, *Anaerostipes*, *Butyrivibrio*, and *Roseburia/Eubacterium rectale* [26, 27].

All qPCR assays were performed in triplicate. Precautions were taken to ensure that the data from each triplicate fell within 0.5 threshold cycle (Ct), and clear outliers (>2 standard deviations) were removed before calculating average Ct of each sample. Melting curves and non-template controls were used to assess run reliability. There was no detectable amplification arising from non-template controls in any of the assays. The amplification efficiencies of all qPCR assays ranged from 91% to 98%.

### Calculation of absolute abundance and copy-number correction

**Fig 2:**
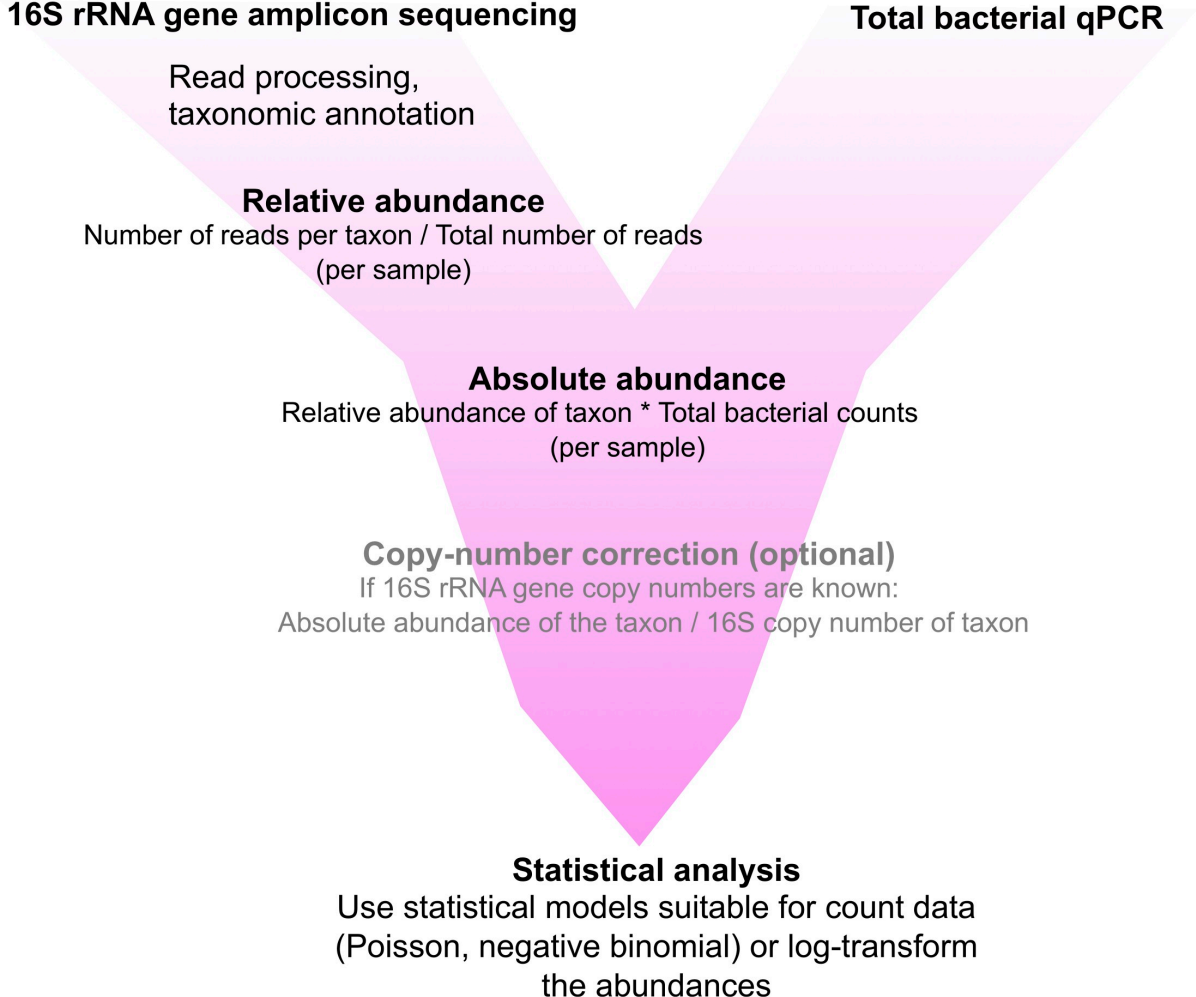
Workflow for implementation of qPCR-based quantitative microbiome profiling. All of these steps are included in R package mare [18].

The sequencing reads assigned to different taxa in each sample were divided by the total number of reads for the sample to obtain relative abundances of the taxa in each sample. The relative abundances obtained based on the sequencing reads were translated into total abundances by multiplying the relative abundance of each taxon by the total bacterial abundance in the sample. These figures were further corrected for 16S rRNA gene copy-number variation by dividing the abundance of a taxon by the number of 16S copies in its genome. For the copy-number correction, we used the 16S copy number database rrnDB [28]. The process is depicted in Fig 2.

## Results and discussion

**Fig 3:**
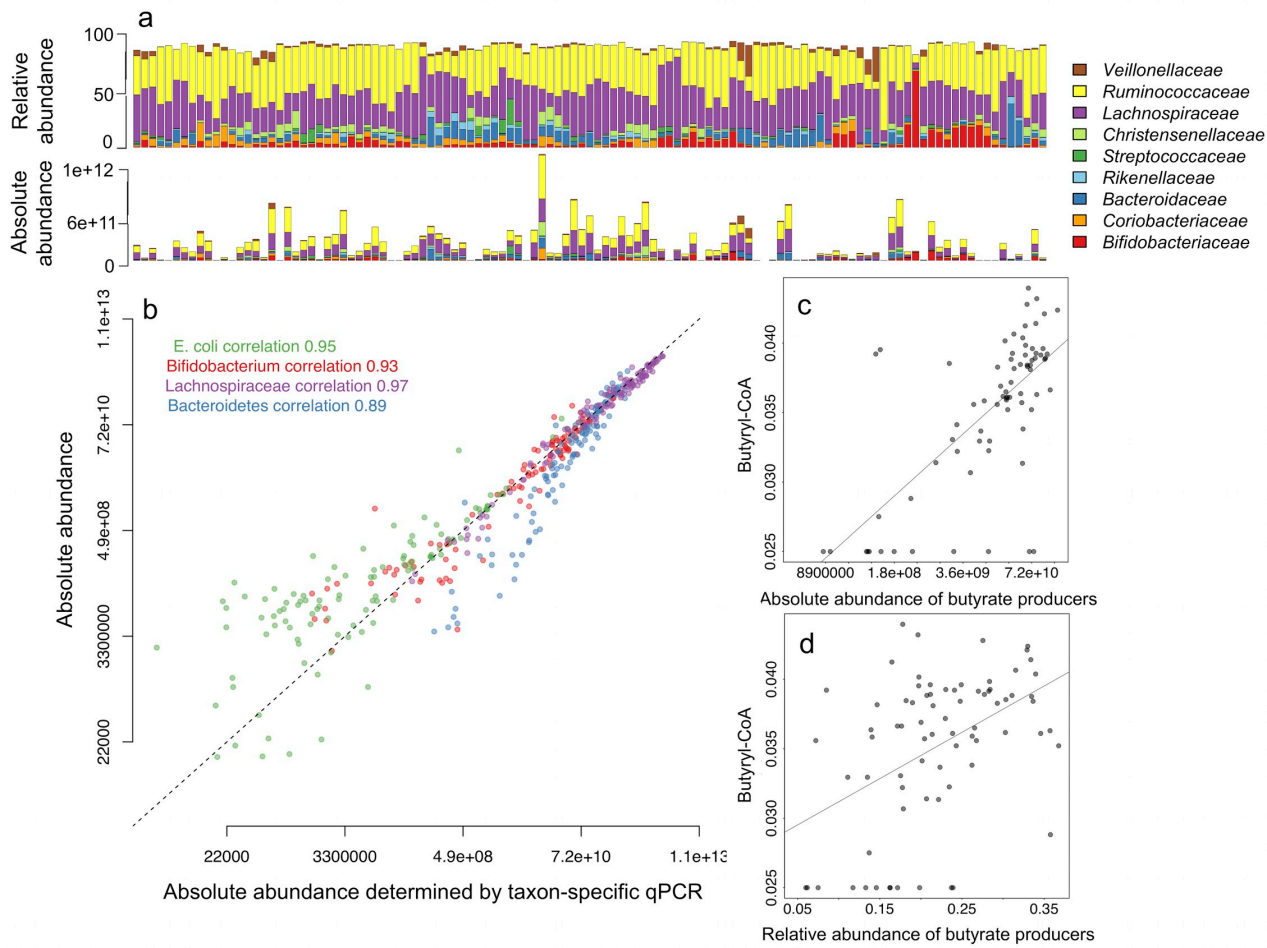
Relative microbiome profiles translated into quantitative microbiome profiles using qPCR. (a) Comparison of relative abundances and estimated absolute abundances of dominant bacterial families in 114 fecal samples. The top panel shows relative abundances based on 16S amplicon sequencing and the lower panels shows the estimated absolute abundances calculated by multiplying the relative abundances with total bacterial load, i.e. qPCR-based estimate of copies of 16S gene per 1 g of feces. (b) Correlation between the qPCR abundances (16S rRNA gene copies per g feces) and the estimated absolute abundances of four taxa representing species, genus, family and phylum levels. The dashed line shows the expected 1:1 correspondence. The correspondence decreases at the very low end of the abundance range, likely due to the relatively lower PCR amplification efficiency and increased stochasticity of the results for low abundance taxa in NGS [30]. The applied library preparation method (dual index TruSeq-tailed 1-step amplification [17]) causes a slight underestimation of Bacteroidetes abundance (unpublished data), explaining the underestimation observed for this phylum compared to qPCR. (c) and (d) show the associations between the qPCR-determined abundance of the butyryl-CoA:acetate CoA-transferase gene and the (c) estimated absolute abundance and (d) relative abundance of butyrate producers detected in the NGS data.

We quantified total bacteria using universal bacterial primers [29] by qPCR in 114 adult fecal DNA samples that have been analyzed for microbiota composition using Illumina MiSeq for 16S rRNA gene amplicon sequencing [14]. The qPCR threshold cycle (Ct) values were converted to the estimates of bacterial genomes present in 1 g of feces as a proxy of total bacterial counts. Absolute abundances of individual taxa can be estimated via multiplying the relative abundances of the NGS-detected taxa by total bacterial counts (Fig 3A). We validated the estimated absolute abundances of four representative taxa by qPCR using taxon-specific primers (S1 Table) for the phylum Bacteroidetes, *Clostridium* cluster XIVa (family *Lachnospiraceae*), genus *Bifidobacterium* and *Escherichia coli* species using standard curve-based absolute quantification. These four taxa were chosen for their representation of different taxonomic ranks and availability of primers and standards. We found near-perfect correlations between the estimated absolute abundances and qPCR abundances in all tested taxa (Fig 3B). By correlating the cumulative absolute abundance of butyrate-producing bacteria to the abundance of the butyryl-CoA:acetate CoA-transferase gene determined by qPCR [24], we show that qPCR-based quantitative microbiome profiling can also be used to more precisely estimate the abundances of specific microbiota functions (Fig 3C and 3D). The estimated absolute abundance of butyrate producers accounted for 47% of the variation in the qPCR-determined butyryl-CoA:acetate CoA-transferase gene abundance (p = 1.15e-11), while the relative abundance explained only 23% (p = 9.92e-06).

Importantly, qPCR-based quantitative microbiome profiling enjoys the following conceptual and practical benefits over other approaches:

1. Cost-effectiveness and feasibility
  qPCR is cost-effective and accessible as the laboratory settings, machinery and reagents are similar to those needed for preparing the NGS libraries. The same DNA extract serves as the starting material both for qPCR and NGS, making qPCR done in 96- or 384-format easy to implement in the workflow for high-throughput analysis of up to thousands of microbiome samples.
2. Simplicity
  qPCR is relatively simple to perform compared to flow cytometry that requires considerable expertise for reproducible results. In fact, flow cytometric enumeration of microbial cells was initially restricted to pure cultures [31] and still remains challenging when performed in complex matrices [32]. Also, no spikes, other exogenous controls, or complicated transformation/computation are needed in qPCR-based quantitative microbiome profiling.
3. Comparability to NGS
  Unlike flow cytometry that counts cells, qPCR and NGS both target bacterial DNA, including extracellular DNA derived from lysed bacteria. Extracellular DNA can be intrinsic or result from the differential lysis of Gram-positive and negative bacteria during the common freeze-thawing prior to fecal DNA extraction. As the 16S profiles from the gut appear very different for intracellular and extracellular DNA [33], qPCR is expected to reflect the NGS-targeted community structure both quantitatively and qualitatively more closely than flow cytometry.
4. Applicability
  qPCR-based quantitative microbiome profiling is applicable also for samples containing a substantial amount of host or non-bacterial DNA, in which bacterial density cannot be reliably estimated by total DNA yield [5]. Moreover, the qPCR-based method can be employed to study also non-bacterial communities where a universal marker gene is available, such as in fungi [12].

It should be noted that relative and absolute abundances based on 16S rRNA gene copies are a proxy for microbial density rather than exact numeration of cells, since 16S rRNA gene copy numbers vary among bacteria. It is, however, possible to computationally correct for 16S rRNA gene copy numbers *post hoc* as we did for this dataset (Fig 2), if the 16S rRNA gene copy numbers of the taxa present in the samples are known. Other potential biases related to PCR-based methods include e.g. inadequate DNA extraction, presence of PCR inhibitors and primer coverage. Nevertheless, these factors play a similar role in the NGS itself [34]. The fact that the qPCR-based approach does not introduce additional biases to those already present in NGS workflows can be thus considered an advantage.

Since several universal bacterial primers have been designed and optimized specifically for qPCR or NGS, it is advisable to consider potential biases resulting from primer-specific amplification efficiency for particular taxa [35] as well as differential primer coverage, when using different primer sets for qPCR and NGS. In this study, we chose a widely-used universal bacterial primer set optimized for qPCR [29] that similarly targets the V3-V4 hypervariable regions as the primer set used for Illumina MiSeq (S1A Table). The qPCR primers have slightly lower but sufficiently comparable coverage for the domain Bacteria compared to the primers for NGS. The coverage of both qPCR and NGS primer sets is highly comparable for the four taxa selected for taxon-specific qPCR (S1B Table), which provides reliable validation of the described method in this study. For future improvement of the herein presented approach, a qPCR assay utilizing exactly the same primer pair as for NGS could be optimized and validated.

One challenge in the cross-study comparability of qPCR-based quantitative microbiome profiling is the reliance on an external qPCR standard from a reference organism required to construct a standard curve. In theory, any typical taxon present in a microbial community of interest can be used as the reference organism for standard curve construction. However, the choice of reference organisms may induce differences in quantification results, as the qPCR amplification efficiencies of different reference organisms may differ [34].

For the statistical analysis of bacterial abundances, relative or absolute, the distribution of the data should be considered. Absolute abundances tend to be greatly right-skewed in distribution, so log-transformation will be useful if statistical methods that assume normal distribution are used. As the abundances are essentially bacterial counts, it is advisable to use statistical tests appropriate for count data, such as generalized linear models with Poisson or negative binomial distribution. For rare taxa with a lot of zeros, zero-inflated models should be considered. Importantly, the right model depends on the distribution of the abundances of a particular taxon, and thus the same model may be not appropriate for all taxa. Notably, this is true also for the analysis of relative abundances. The R package *mare* [18] can handle both types of data, automatically selecting the suitable statistical model for each taxon.

## Conclusions

In conclusion, we caution against the analysis of microbiome NGS data solely relying on relative abundance, since compositionality may skew biological inferences from microbiome studies *per* our simulation data as well as the previously published studies. Although relative taxon abundance can be indicative, absolute quantification is necessary for obtaining a comprehensive understanding of the dynamics and interactions of the microbiome. To this end, we suggest qPCR-based quantitative microbiome profiling be integrated in standard NGS-based microbiome analysis.

## Supporting information

S1 Fig Selected results of a simulated intervention in a complex community (91 taxa). (DOCX)

S2 Fig Results of a simulated intervention in a simple community (10 taxa). (DOCX)

S1 Table List of primers used in this study. (PDF)

S1 File Raw data in spreadsheet format. This file contains the following data: simulated intervention used in S1 and S2 Figs, total bacterial and taxon-specific qPCR, and qPCR of butyryl-CoA:acetate CoA-transferase gene.
(XLSX)
