# Supplementary material for: Quantitative PCR provides a simple and accessible method for quantitative microbiota profiling
Source: PLoS One. 2020 Jan 15;15(1):e0227285. doi: 10.1371/journal.pone.0227285 (PMC6961887; doi:10.1371/journal.pone.0227285)
Supplement: S1 Fig — (DOCX) [file pone.0227285.s001.docx]

Control group

Intervention group

**a** group


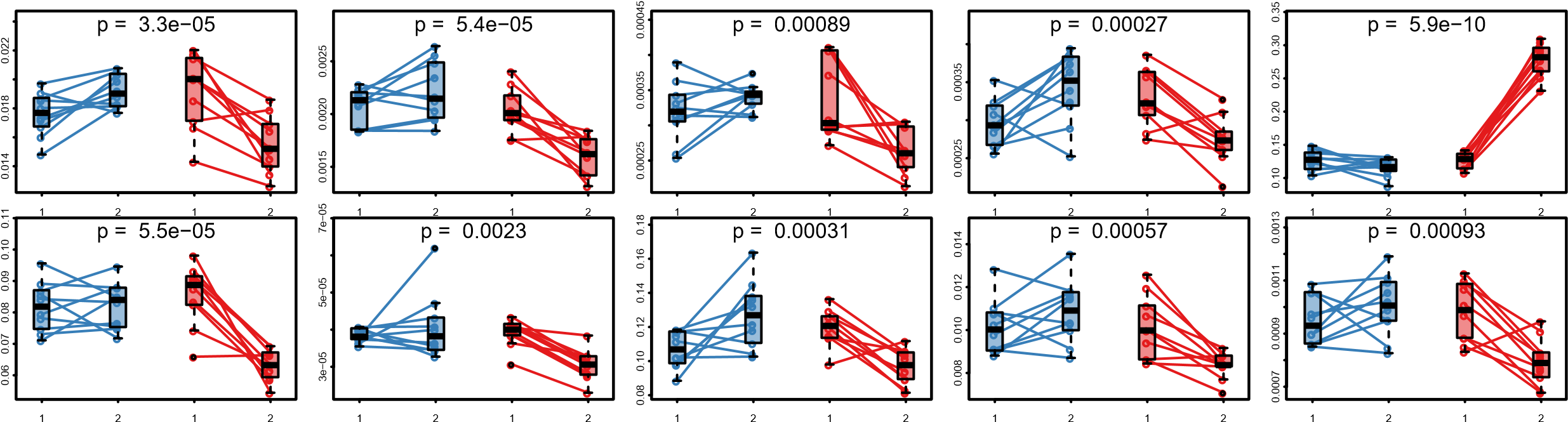


**b**


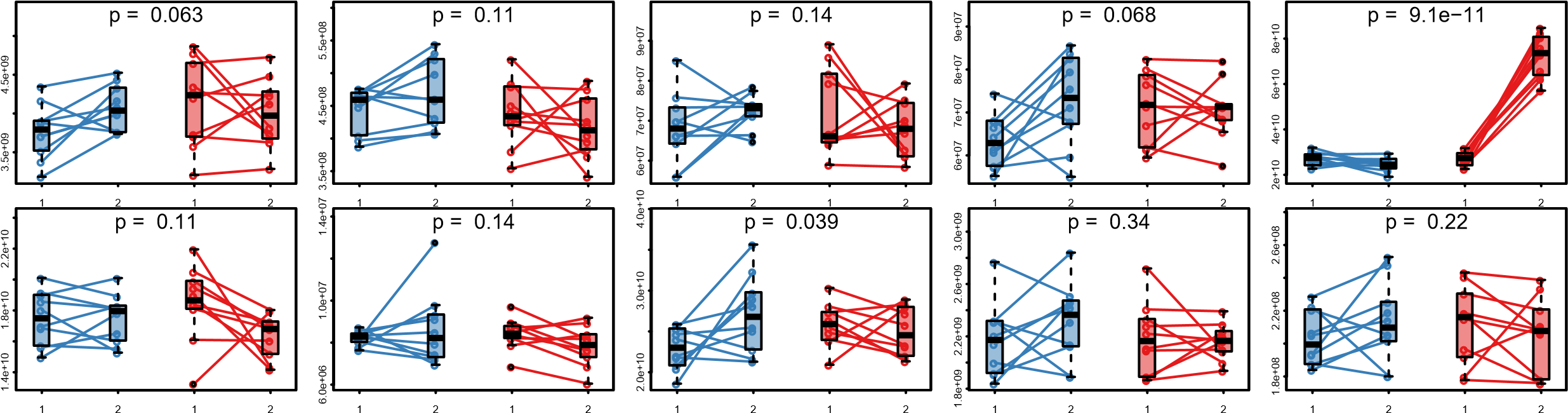


**S1 Fig. Selected results of a simulated intervention in a complex community (91 taxa).**

To demonstrate the effect of compositionality on interpretation of microbiome NGS data, another intervention was simulated where one single taxon was increased in abundance in the treatment group (n=10) and nothing changed in the control group (n=10). The panels show the 10 most significantly affected taxa (significantly different change between the control and the treatment group) based on relative abundance in the simulated intervention. Each box shows one taxon, in the same order for relative (a) and absolute (b) data. The change in the abundance of each taxon from baseline to the post-intervention sample was calculated for each individual and the significance of the difference between the treatment and the control groups was tested using analysis of variance (ANOVA). (a) Relative data. While only one taxon was actually affected by the intervention, 62 out of 91 taxa (67%) were significantly different between the groups at p-value cut-off 0.05, and 19 taxa (21%) at p-value cut-off 0.005. (b) Absolute data. While one taxon was affected by the intervention, additionally only 3 false positives (3%; not among the shown top 10 taxa based on the relative data) were identified with p-value cut-off 0.05 when analyzing the absolute abundances due to random variation added in the simulation, and no false positives were detected at the 0.005 level.
