# Supplementary material for: Quantitative PCR provides a simple and accessible method for quantitative microbiota profiling
Source: PLoS One. 2020 Jan 15;15(1):e0227285. doi: 10.1371/journal.pone.0227285 (PMC6961887; doi:10.1371/journal.pone.0227285)
Supplement: S2 Fig — (DOCX) [file pone.0227285.s002.docx]

Control group

Intervention group

**a**


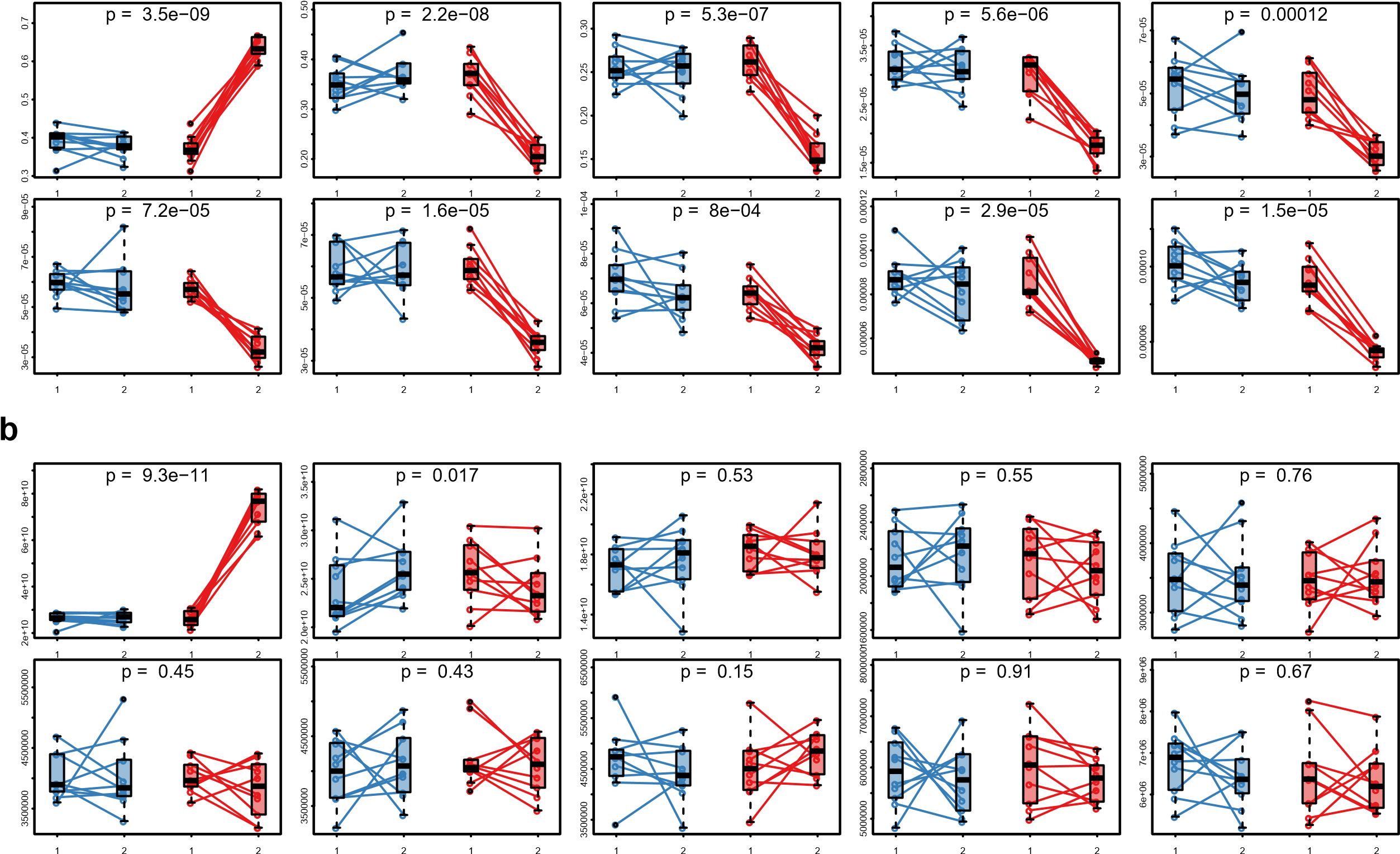


**S2 Fig. Results of a simulated intervention in a simple community (10 taxa).**

In the same simulated intervention as S1 Fig., the compositionality problem was even more debilitating when the community is simple. (a) When the relative abundance data were analyzed, all taxa appeared significantly affected by the intervention at strict p-value cut-off 0.005. (b) Absolute abundance data show correctly that only one taxon was actually affected by the intervention (p-value < 0.005). No false positives were detected.
