# Supplementary material for: Quantitative PCR provides a simple and accessible method for quantitative microbiota profiling
Source: PLoS One. 2020 Jan 15;15(1):e0227285. doi: 10.1371/journal.pone.0227285 (PMC6961887; doi:10.1371/journal.pone.0227285)
Supplement: S1 Table — (PDF) [file pone.0227285.s003.pdf]

S1 Table. List of primers and PCR conditions used in this study.

S1 Table A.

Target	Primer sequence (5'-3')	Amplicon size	Mg (mM)	Annealing (°C)	Detection (°C)	Amount of template DNA for	Standard/reference organism for qPCR	Coverage (%)*	Original reference
Bacteria (qPCR)	F: 5'-TCCTACGGGAGGCAGCAGT-3'<br>R: 5'-GGACTACCAGGGTATCTAATCCTGTT-3'	466	3	50	82	0.5	*B. longum*	83.1**	[1]
Bacteria (16S rRNA gene V3-V4 amplicon sequencing)	F: 5'-CCTACGGGNGGCWGCAG-3'<br>R: 5'-GACTACHVGGGTATCTAATCC-3'	444	-	-	-	-	-	95**	[2]
Bacteroides-Prevotella-Porphyromonas	F: 5'-GGTGTCGGCTTAAGTGCCAT-3'<br>R: 5'-CGGAYGTAAGGGCCGTGC-3'	140	3	68	80	0.5	*B. fragilis*	95.8	[3]
Bifidobacterium spp.	F: 5'-TCGCGTCYGGTGTGAAAG-3'<br>R: 5'-CCACATCCAGCRTCCAC-3'	243	3	58	85	25	*B. longum*	93.9	[3]
Clostridium coccoides-Eubacterium rectale group (*Lachnospiraceae*)	F: 5'-CGGTACCTGACTAAGAAGC-3'<br>R: 5'-AGTTTYATTCTTGCGAACG-3'	429	4	55	85	0.5	*R. productus*	85.9	[3]
Escherichia coli subgroup (*E. coli*, *Hafnia alvei* and *Shigella* spp.)	F: 5'-GTTAATACCTTTGCTCATTGA-3'<br>R: 5'-ACCAGGGTATCTAATCCTGTT-3'	340	3	60	85	25	*Escherichia coli DSM 6897*	90.6	[4]
Butyryl-CoA CoA transferase	F: 5'-GCIGAICATTTCACITGGAAYWSITGGCAYATG-3'<br>R: 5'-CCTGCCTTTGCAATRTCIACRAANGC-3'	530	3	53	82	25	-	-	[5]

*The coverage (i.e. the percentage of potentially covered taxa within the target) was estimated using the SILVA TestPrime tool with the sSU-NR database version 132, allowing one mismatch during primer annealing to simulate realistic PCR behavior [2].
**Detailed breakdown of coverage for the targets at lower taxonomic levels is listed in S1 Table B.

S1 Table B.

Primers set	Target	Coverage (%)
Bacteria (qPCR)	*Bacteroides-Prevotella-Porphyromonas*	93.7
	Bifidobacterium spp.	93.7
	Clostridium coccoides-Eubacterium rectale group (*Lachnospiraceae*)	95
	Escherichia coli subgroup (*E. coli*, *Hafnia alvei* and *Shigella* spp.)	94
Bacteria (16S rRNA gene V3-V4 amplicon sequencing)	*Bacteroides-Prevotella-Porphyromonas*	95.7
	Bifidobacterium spp.	94.1
	Clostridium coccoides-Eubacterium rectale group (*Lachnospiraceae*)	95.9
	Escherichia coli subgroup (*E. coli*, *Hafnia alvei* and *Shigella* spp.)	95.1

References

1. Nadkarni MA, Martin FE, Jacques NA, Hunter N. Determination of bacterial load by real-time PCR using a broad-range (universal) probe and primers set. Microbiology (Reading, England). 2002;148(Pt 1):257-66. Epub 2002/01/10. doi: 10.1099/00221287-148-1-257. PubMed PMID: 11782518.

2. Klindworth A, Pruesse E, Schweer T, Peplies J, Quast C, Horn M, et al. Evaluation of general 16S ribosomal RNA gene PCR primers for classical and next-generation sequencing-based diversity studies. Nucleic acids research. 2013;41(1):e1. Epub 2012/08/31. doi: 10.1093/nar/gks808. PubMed PMID: 22933715; PubMed Central PMCID: PMCPMC3592464.

3. Rinttila T, Kassinen A, Malinen E, Krogius L, Palva A. Development of an extensive set of 16S rDNA-targeted primers for quantification of pathogenic and indigenous bacteria in faecal samples by real-time PCR. Journal of applied microbiology. 2004;97(6):1166-77. Epub 2004/11/18. doi: 10.1111/j.1365-2672.2004.02409.x. PubMed PMID: 15546407.

4. Malinen E, Kassinen A, Rinttila T, Palva A. Comparison of real-time PCR with SYBR Green I or 5'-nuclease assays and dot-blot hybridization with rDNA-targeted oligonucleotide probes in quantification of selected faecal bacteria. Microbiology (Reading, England). 2003;149(Pt 1):269-77. Epub 2003/02/11. doi: 10.1099/mic.0.25975-0. PubMed PMID: 12576600.

5. Louis P, Flint HJ. Development of a semiquantitative degenerate real-time pcr-based assay for estimation of numbers of butyryl-coenzyme A (CoA) CoA transferase genes in complex bacterial samples. Applied and environmental microbiology. 2007;73(6):2009-12. Epub 2007/01/30. doi: 10.1128/aem.02561-06. PubMed PMID: 17259367; PubMed Central PMCID: PMCPMC1828812.
